# Co-delivery of nitric oxide and antibiotic using polymeric nanoparticles†
†Electronic supplementary information (ESI) available: NMR spectra, SEC traces, FTIR, UV-Vis spectra, NO release using amperometric measurement (Fig. S1–S11) are available free of charge *via* the internet. See DOI: 10.1039/c5sc02769a



**DOI:** 10.1039/c5sc02769a

**Published:** 2015-11-10

**Authors:** Thuy-Khanh Nguyen, Ramona Selvanayagam, Kitty K. K. Ho, Renxun Chen, Samuel K. Kutty, Scott A. Rice, Naresh Kumar, Nicolas Barraud, Hien T. T. Duong, Cyrille Boyer

**Affiliations:** a Centre for Advanced Macromolecular Design (CAMD) and Australian Centre for NanoMedicine (ACN) , School of Chemical Engineering , UNSW Australia , Sydney , NSW 2052 , Australia . Email: cboyer@unsw.edu.au ; Email: hien.duong@sydney.edu.au; b School of Chemistry , UNSW Australia , Sydney , NSW 2052 , Australia; c Centre for Marine-Innovation , School of Biological , Earth and Environmental Sciences , University of New South Wales , Sydney , Australia 2052 . Email: n.barraud@melix.org; d The Singapore Centre for Environmental Life Sciences Engineering and The School of Biological Sciences , Nanyang Technological University , Singapore; e Department of Microbiology , Genetics of Biofilms Unit , Institute Pasteur , Paris , France

## Abstract

The rise of hospital-acquired infections, also known as nosocomial infections, is a growing concern in intensive healthcare, causing the death of hundreds of thousands of patients and costing billions of dollars worldwide every year. In addition, a decrease in the effectiveness of antibiotics caused by the emergence of drug resistance in pathogens living in biofilm communities poses a significant threat to our health system. The development of new therapeutic agents is urgently needed to overcome this challenge. We have developed new dual action polymeric nanoparticles capable of storing nitric oxide, which can provoke dispersal of biofilms into an antibiotic susceptible planktonic form, together with the aminoglycoside gentamicin, capable of killing the bacteria. The novelty of this work lies in the attachment of NO-releasing moiety to an existing clinically used drug, gentamicin. The nanoparticles were found to release both agents simultaneously and demonstrated synergistic effects, reducing the viability of *Pseudomonas aeruginosa* biofilm and planktonic cultures by more than 90% and 95%, respectively, while treatments with antibiotic or nitric oxide alone resulted in less than 20% decrease in biofilm viability.

## Abstract 2

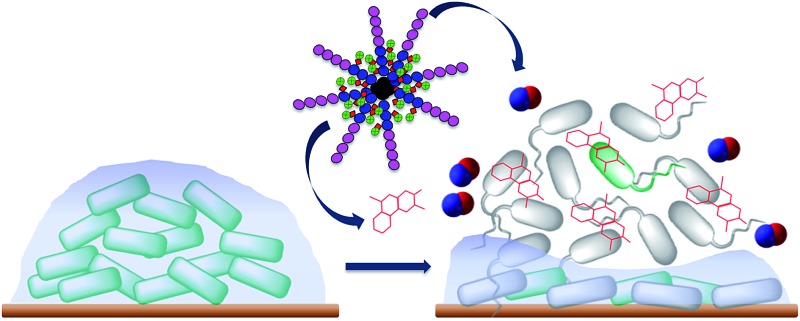
The rise of hospital-acquired infections, also known as nosocomial infections, is a growing concern in intensive healthcare, causing the death of hundreds of thousands of patients and costing billions of dollars worldwide every year.

## Introduction

Nosocomial infections are the fourth leading cause of disease in the U.S.A. and Europe with over 3.5 million cases annually,^1^ resulting in significant increases in healthcare costs. More importantly, the number of cases (and by consequence deaths) is rapidly increasing due to the emergence of bacteria resistant to antibiotics.^2,3^ One key adaptive process used by bacteria that leads to their survival and development of resistance after antibiotic treatments is the ability to form multicellular communities of cells encased in a matrix of secreted polymeric substances known as microbial biofilms.^4^ Their formation and persistence have a considerable impact for patient health, as many biofilm infections are difficult to resolve, and often result in chronic or recurrent infections.^5–7^ Indeed, bacteria in biofilms show significantly increased resistance to external stresses, including antimicrobials and host immune defenses, compared with free-living single bacterial cells.^8,9^ Biofilms can also favor gene transfer between bacteria, thus spreading antibiotic resistance or converting a previously non-virulent commensal organism into a virulent pathogen.^10^ Consequently, biofilm infections present a number of clinical challenges,^11–17^ including diseases involving uncultivable species, chronic inflammation, impaired wound healing, rapidly acquired antibiotic resistance, and the spread of infections. Accordingly, there is an urgent need for novel therapeutics and treatment strategies that are effective against biofilms and biofilm-related infections.

Biofilm researchers have now established that most bacteria follow a lifecycle in which the biofilm mode of growth is the main phase. Bacterial cells can alternate between the biofilm and the planktonic lifestyles *via* transition stages of either attachment or dispersal that involve the expression of specific genes and are highly regulated.^18^ In 2006, the biologically ubiquitous nitric oxide (NO) gas was found to be a major signal for biofilm dispersal in the important human pathogen *Pseudomonas aeruginosa*,^19,20^ which was found to account for up to 30% of hospital-acquired infectious diseases (nosocomial).^21^ Follow-up studies showed that exposure to NO in the pM and low nM range can induce dispersal in several other single- and multi-species bacterial and yeast biofilms and that the effects correlate with increases in bacterial phosphodiesterase activity and associated decreases in intracellular levels of the secondary messenger cyclic di-GMP.^22,23^ After the onset of dispersal induced by NO, both released cells and the remaining biofilms display enhanced sensitivity towards a range of antibiotics, including aminoglycosides.^19,24,25^ Furthermore, it was recently shown for the first time that inducing a full-scale dispersal event, by means of a genetically modified organism, can clear infections in animal models without killing the host.^26^ Similar results were obtained *in vitro* using NO donor compounds, which under a specific condition release NO gas. Therefore, all these combined results have unveiled a new anti-biofilm strategy, which uses low concentrations of NO-donor compounds in combination with antibiotics to eradicate bacterial biofilm infections.^27^

However, NO donors present a poor stability in biological media, resulting in fast release of NO, which severely limits any potential applications. To overcome these problems, the design of NO donor specifically by enzymatic reactions and the encapsulation of NO donor into polymeric nanoparticles or inorganic materials have been proposed by our group and others.^24,28–41^ It is well known that the encapsulation of therapeutic compounds into nanoparticles enhances their stability and solubility as well as increases their local concentration.^42–45^ In previous studies, we and others have developed polymeric and organic/inorganic nanoparticles for the delivery of NO, facilitating its application in dispersion or eradication of biofilms.^28,46–48^ For instance, we made core cross-linked star polymers containing *N*-diazeniumdiolate (NONOate) compounds that were capable of releasing NO in a controlled manner for several days, and these polymeric materials were able to prevent and disperse biofilms.^48^ In addition, NO at high concentration (typically mM) can have a killing effect on several types of bacteria as demonstrated by Schoenfisch and co-workers.^46,49–54^ The authors have investigated a range of nano-scaled objects with various shapes, and studied their efficacy as bactericidal agents.^53^ Very recently, the combination of cationic polymers presenting antimicrobial activity with NO for the treatment of biofilms was reported in recent papers by Schoenfisch,^55,56^ significantly enhanced the killing ability of the antibacterial polymers.

In this study, we combined in one polymeric nanoparticle a NO donor and an aminoglycoside antibiotic, gentamicin. More importantly, in this approach, the NO donor was directly obtained by reaction of gentamicin with NO gas to yield gentamicin-NONOate complex. By engineering the nanoparticles (*i.e.* placing gentamicin-NONOate in the core), we aimed to obtain a simultaneous and sustainable release of gentamicin and NO, with both released agents acting synergistically on biofilms. The gentamicin-NONOate nanoparticles were found to effectively disperse biofilms of the model organism *P. aeruginosa*, and, at concentrations of 10–50 μM, strongly decreased the viability of both biofilm and planktonic cells by more than 90% and 95%, respectively. In contrast, gentamicin and NO donor separately presented a lower efficiency against biofilm and planktonic cells.

## Experiments and methods

### Materials

All chemicals were used as received from Ajax and Sigma-Aldrich, unless otherwise specified. Monomer oligo(ethylene glycol) methyl ether methacrylate with an average *M*_n_ of 300 g mol^–1^ (OEGMA) and 3-vinylbenzaldehyde (VBA) were de-inhibited by passing them through a column of basic alumina. 2,2′-Azobisisobutyronitrile (AIBN) was purified by recrystallization from methanol.

### Synthesis of POEGMA macro-RAFT agent

The RAFT agent, 4-cyanopentanoic acid dithiobenzoate (CPADB) was prepared according to a published procedure.^57^ OEGMA (2.58 g, 8.60 × 10^–3^ mol), CPADB RAFT agent (5.33 × 10^–2^ g, 1.91 × 10^–4^ mol) and AIBN (6.27 × 10^–3^ g, 3.82 × 10^–5^ mol) were dissolved in 20 mL of toluene in a round-bottom flask equipped with a magnetic stirrer bar. The flask was then sealed with a rubber septum and purged with nitrogen gas for 30 min. The reaction mixtures were then immersed in a preheated oil bath at 70 °C. After 17 h, the polymerization was terminated by quenching the samples in an ice bath for 5 min. The POEGMA polymer was purified three times by precipitation with excess petroleum spirits (boiling range of 40–60 °C) followed by centrifugation (7000 rpm for 15 min) and the polymer was dried under vacuum at room temperature. The samples were stored at 4 °C until required for further chain extension. By comparing the intensity of vinyl proton peaks (6.1 and 5.6 ppm) to that of ester –OC*H*_2_ proton peaks (4.1 ppm), the conversion of monomer during the course of polymerization was determined using ^1^H NMR. After 17 h, a conversion of 80% was obtained. The molecular weight of the POEGMA macro-RAFT agent was measured to be 11 200 g mol^–1^ (PDI = 1.08) by DMAC SEC and *M*_n, NMR_ = 10 800 g mol^–1^ by ^1^H NMR.

### Synthesis of POEGMA-*b*-PVBA

POEGMA with 36 repeating units (*M*_n, NMR_ = 10 800 g mol^–1^, *M*_n, SEC_ = 11 200 g mol^–1^) was used as a macro-RAFT agent for chain extension with VBA. The number of repeating units of POEGMA was calculated from the monomer conversion obtained from ^1^H NMR. The POEGMA macro-RAFT agent (1 g, 9.25 × 10^–5^ mol) was dissolved in 5 mL of toluene containing VBA (1.84 × 10^–1^ g, 1.39 × 10^–3^ mol). The reaction mixture was purged with nitrogen gas for 30 min in an ice bath. The polymerization was carried out in an oil bath at 70 °C overnight. The polymerization was terminated by placing the samples on ice for 5 min. The POEGMA polymer was purified three times by precipitation in excess of diethyl ether followed by centrifugation (7000 rpm for 15 min), and the polymer was then dried under reduced pressure at room temperature. Block copolymer with 36 repeating units of OEGMA, 7 repeating units of VBA (as confirmed by ^1^H NMR in ESI, Fig. S1†) was chosen for further conjugation with gentamicin (*M*_n, theo_ = 11 700 g mol^–1^, *M*_n, SEC_ = 13 700 g mol^–1^).

### Conjugation of POEGMA-*b*-PVBA to gentamicin

Gentamicin sulfate (Enzo Life Sciences, Sapphire Bioscience Pty. Ltd., Australia) (0.3 g, 2.02 × 10^–4^ mol) and 100 μL triethylamine (TEA) were dissolved in 2.5 mL of distilled water. The solution was left in an incubator at 37 °C whilst being shaken at 140 rpm for 1 h. Upon completion, drug solution was added into 2.5 mL of POEGMA-*b*-PVBA (0.3 g, 2.04 × 10^–5^ mol) in distilled water and the mixture of polymer and drug was incubated at 37 °C with shaking at 100 rpm for a further 48 h. The mixture was then precipitated in acetonitrile and centrifuged at 7500 rpm for 5 min to remove unreacted gentamicin and salt formed. The supernatant was collected and the precipitation step in acetonitrile was repeated three times. Anhydrous magnesium sulfate was used as a drying agent to remove water from the mixture for further reaction with NO gas to introduce the NO-releasing NONOate moiety to the polymer–drug conjugates in acetonitrile.

### Attachment of NONOate to conjugated POEGMA-*b*-PVBA with gentamicin

The conjugated POEGMA-*b*-PVBA with gentamicin (0.3 g) was dissolved in acetonitrile (5 mL) and placed in a Parr apparatus and clamped. The apparatus was then purged and evacuated with nitrogen three times and pressurized to 5 atm NO at 25 °C for 48 h to form NONOate NO donors. Excess NO was then vented through purging with nitrogen gas. The NONOate polymer was then stored at 4 °C until required for further analysis.

### Determination of NO release by Griess assay and amperometric measurement

NO released from the polymer at specified time intervals was determined using a standard Griess reagent kit (G-7921, Molecular Probes), which is normally used for nitrite determination. NONOate readily releases NO upon contact with water at physiological pH. Typically, 10 mg gentamicin-NONOate containing polymer sample was dissolved in 2 mL of phosphate-buffered saline (PBS). The solution was enclosed in a sealed dialysis membrane (Cellu-Sep 3500 MWCO) that allows free diffusion of NO. The membrane was then immersed in a 6 mL PBS solution and incubated at 37 °C for up to 24 h. At various time points, a 100 μL aliquot from the PBS solution was taken for determining concentration of NO. Since NO readily oxidises to nitrite and nitrate upon contact with water, first the reduction of nitrate to nitrite was conducted through a nitrate reductase. For each 100 μL of sample, 12.5 μL of nitrate reductase and 12.5 μL of enzyme cofactor were added into the solution and incubated at room temperature for 30 min. Then, 120 μL of Griess reagents was added to the sample and left to incubate at room temperature for 30 min. The sample was then topped up with 395 μL of distilled water to make up a total volume of 640 μL. The preparation procedure was repeated for samples at different time points. The UV-Vis absorbance of the resulting solutions was determined at 548 nm and the total nitrite concentration in the sample solutions at different time points were calculated from a standard curve and converted to cumulative NO release.

NO was detected amperometrically by using a TBR4100 free radical analyzer with Lab-Trax-4 digital recorder (World Precision Instruments, Sarasota, USA) and fitted with an NO specific sensor (ISO-NOP). The NO sensor, which was freshly calibrated using *S*-nitroso-*N*-acetylpenicillamine (SNAP) and copper sulfate according to the manufacturer's instructions, was immersed in a vial containing 10 mL PBS (pH 7.4) and continuously stirred at 37 °C. After the baseline had stabilized, 100 μL of 100 mM gentamicin-NONOate containing polymer solution was added into the vial and instantaneous NO levels were monitored over 3.5 h. After this time, 50 μL of a 50 mM solution of the free radical scavenger 2-phenyl-4,4,5,5-tetramethylimidazoline-1-oxyl-3-oxide (PTIO) was injected into the vial in order to confirm that the amperometric signals being observed were due to NO.

### Analytical instruments

#### ^1^H-NMR spectroscopy

Monomer conversions and polymer compositions were analyzed by ^1^H-NMR using a Bruker AC300F (300 MHz) spectrometer and a Bruker DPX300 (300 MHz) spectrometer.

OEGMA monomer conversion was determined *via* ^1^H-NMR spectroscopy by the following equation: *α* ^OEGMA^ = 1 – (∫_5.6 ppm_/(∫_4.1 ppm_/2)), where ∫ is the peak integral of monomer (vinyl proton at 5.6 ppm, 1H) and the polymer (ester proton at 4.1 ppm, 2H).

The experimental *M*_n, NMR_ was calculated by using the dithiobenzoate end group peak (*i.e.*7.8 ppm) in the ^1^H-NMR as a reference, as follows:

*M*_n, NMR_ = (∫_4.1 ppm_/2)/(∫_7.8 ppm_) × *M*_w, OEGA_ + *M*_w, CPADB_. ∫_4.1 ppm_ and ∫_7.8 ppm_ represent the peak integral of OEGMA peak at 4.1 ppm (2H) and the dithiobenzoate peak (1H) at 7.8 ppm, respectively. *M*_w, OEGMA_ and *M*_w, CPADB_ represent the molar mass of OEGMA and CPADB, respectively.

VBA conversion was calculated from ^1^H NMR spectrum of the reaction mixture using the following equation: *α* ^OEGMA^ = ∫_9.8 ppm_/(∫_10.0 ppm_ + ∫_9.8 ppm_), where ∫_9.8 ppm_ and ∫_10.0 ppm_ correspond to the integrals of aldehyde protons of poly(vinylbenzaldehyde) and vinyl benzaldehyde monomer, respectively.

NMR molecular weight was calculated according to *M*_n, NMR_= ((∫_9.8 ppm_/(∫_4.1 ppm_/2)) × DP_n_^OEGMA^) × *M*_w, VBA_ + *M*_n, POEGMA macroRAFT_, where *M*_w, VBA_ and *M*_n, POEGMA macroRAFT_ are the molecular weight of monomer and macro RAFT agent, respectively.

In addition, ^1^H NMR spectroscopy was used to demonstrate the conjugation of gentamicin drug to polymers as well as its release in acidic and neutral media, by monitoring changes in the signal at 9.8 ppm.

#### Size exclusion chromatography (SEC)

SEC analyses of polymer samples were performed in *N*,*N*′-dimethylacetamide [DMAc with 0.03% w/v LiBr and 0.05% 2,6-di-butyl-4-methylphenol (BHT)] at 50 °C at flow rate of 1 mL min^–1^ with a Shimadzu modular system comprising an SIL-10AD automatic injector, a Polymer Laboratories 5.0 μL bead-size guard column (50 × 7.8 mm) followed by four linear PL (Styragel) columns (10^5^, 10^4^, 10^3^ and 500 Å) and an RID-10A differential refractive-index detector. The SEC calibration was performed with narrow-polydispersity polystyrene standards ranging between 104 and 2 000 000 g mol^–1^. Polymer solutions at 2–3 mg mL^–1^ were prepared in the eluent and filtered through 0.45 μm filters prior to injection.

#### Attenuated total reflectance-Fourier transform infrared spectroscopy (ATR-FTIR)

ATR-FTIR measurement of samples was performed using a Bruker IFS66/S Fourier transform spectrometer by averaging 128 scans with a resolution of 4 cm^–1^. Polymer samples were pre-dried as thin films for ATR-FTIR analysis.

#### Dynamic light scattering (DLS)

DLS measurements were performed using a Malvern Zetasizer Nano Series running DTS software (4 mW, He–Ne laser, *λ* = 633 nm) and an avalanche photodiode (APD) detector. The scattered light was measured at an angle of 175° for DLS measurements. The temperature was stabilized to ±0.1 °C of the set temperature. All samples were prepared in MilliQ water at the concentration of ∼0.2 mg mL^–1^ of polymer and filtered through a 0.45 μm pore size filter to remove dust prior to measurement.

#### UV-Visible spectroscopy

UV-Vis spectra were recorded in a quartz cuvette using a CARY 3000 spectrometer from Bruker at 25 °C.

#### Transmission electron microscopy (TEM)

Nanoparticles size and morphologies were measured and analyzed using a JEOL 1400 transmission electron microscope at an accelerating voltage of 80 kV. A drop of samples solution was deposited onto a formwar-coated copper grid and the water was evaporated under air. No staining was applied.

#### Elemental analysis using X-ray photoelectron spectrometer (XPS)

A Kratos Axis ULTRA XPS incorporating a 165 mm hemispherical electron energy analyzer was used. The incident radiation was monochromatic A1 X-rays (1486.6 eV) at 225 W (15 kV, 15 ma). Survey (wide) scans were taken at an analyzer pass energy of 160 eV and multiplex (narrow) higher resolution scans at 20 eV. Survey scans were carried out over 1200–0 eV binding energy range with 1.0 eV steps and a dwell time of 100 ms. Narrow higher resolution scans were run with 0.2 eV steps and 250 ms dwell time. Base pressure in the analysis chamber was 1.0 × 10^–9^ Torr and during sample analysis 1.0 × 10^–8^ Torr. The data were analyzed by the software XPS PEAK. An integral (nonlinear) backgrounds subtraction was used for the treatment of XPS data. The peak shape assumption uses the asymmetric mixed Gaussian–Lorentzian functions.

### Biofilm dispersal and killing assays

The laboratory strain *P. aeruginosa* PAO1 was used to characterize the effects of NO and/or antibiotic conjugated polymers on biofilm formation. Biofilms were grown as previously described^40,58^ with some modifications. Briefly, in all assays, overnight cultures in Luria Bertani medium were diluted to an OD_600_ of 0.005 in 1 mL M9 minimal medium (containing 48 mM Na_2_HPO_4_, 22 mM KH_2_PO_4_, 9 mM NaCl, 19 mM NH_4_Cl, 2 mM MgSO_4_, 20 mM glucose, 100 μM CaCl_2_, pH 7.0) in tissue-culture treated 24-well plates (Costar, Corning®). The plates were incubated at 37 °C with shaking at 180 rpm in an orbital shaker (model OM11, Ratek, Boronia, Australia) and the biofilms were allowed to grow for 6 h without any disruption.

At this time, various treatments, including gentamicin-NONOate (GEN-NO) nanoparticles, free gentamicin or the NO donor *N*-[4-[1-(3-aminopropyl)-2-hydroxy-2-nitrosohydrazino]butyl]-1,3-propanediamine (spermine NONOate) (Cayman Chemical, USA), which has a half-life of ∼39 min at 37 °C,^59,60^ at different concentrations as indicated, were added to the wells. Each treatment was added from a 10 μL aliquot of a stock solution at the appropriate concentration of the compound dissolved in 10 mM NaOH and previously sterilized by passing through a 0.22 μm pore size filter. The plates were incubated for a further 1 h or 2.5 h before quantifying the biomass or viability of both planktonic and biofilm bacteria.

Biofilm biomass was determined by crystal violet staining. The biofilm on the well surfaces was first washed once with 1 mL of PBS, before adding 0.03% crystal violet stain made from a 1 : 10 dilution of Gram crystal violet (BD) in PBS. The plates were incubated on the bench for 20 min before washing the wells twice with PBS. Photographs of the stained biofilms were obtained using a digital camera. The amount of remaining crystal violet stained biofilm was quantified by adding 1 mL 100% ethanol and measuring OD_550_ of the homogenized suspension by using a microtitre plate reader (Wallac Victor^2^, Perkin-Elmer). OD measurements of control wells where no bacteria were added at the beginning of the experiment were subtracted from all values (*i.e.* OD_550_ = 0.10).

For viability measurements, the BacTiter-Glo Microbial Cell Viability Assay (Promega, Alexandria, Australia), which is based on quantitation of the ATP present in bacteria by using a thermostable luciferase and is known to correlate to viable cell counts, was used.^61^ After the final 1 h or 2.5 h incubation with various treatments, the planktonic solution was directly mixed with the BacTiter-Glo reagent following the manufacturer's instructions and after 5 min incubation, and the luminescence was measured by using a multimode microtitre plate reader (Wallac Victor^2^, Perkin-Elmer). In order to measure the viability of biofilm bacteria, biofilms on the interior surfaces of the wells were first washed twice with PBS before being re-suspended and homogenized in PBS by incubating in an ultrasonication bath (150 W, 40 kHz; Unisonics, Australia) for 20 min. This resuspension method is used similarly for analyzing colony-forming units (CFU) from biofilms.^58^ Re-suspended biofilm cells were then mixed with BacTiter-Glo reagent and their viability quantified by luminescence measurement as described above.

### Confocal microscopy analysis

For microscopy analysis, *P. aeruginosa* biofilms were grown in glass-bottom, 24-well plates (MatTek Corporation, Ashland MA, USA) as described above. After 7 h incubation including 1 h treatment, biofilms were rinsed twice with PBS before being stained with LIVE/DEAD® *Bac*Light™ bacterial viability kit reagents (L-7007, Molecular Probes) according to the manufacturer's procedure. One microliter of each of the two components were mixed thoroughly in 1 mL of PBS, then 0.3 mL of this solution was trapped between the sample and the glass microscopy slide and allowed to incubate at room temperature in the dark for 20 min. The samples were observed with an Olympus FV1000 Confocal Inverted Microscope, and imaged with a Leica DFC 480 camera. Cells that were stained green were considered to be viable, those that stained red and stained both green and red were considered to be non-viable.

### Statistical analysis

All assays included 2 replicates and were repeated in 2 independent experiments. Statistical analyses were performed with GraphPad Prism 6 (GraphPad Software) using one-way ANOVA followed by Dunnett's multiple comparison test comparing treated samples to the untreated control.

## Results and discussion

In this study, we designed polymeric nanoparticles for the co-delivery of nitric oxide and an antibiotic, gentamicin. To achieve a controlled release of gentamicin, we conjugated gentamicin to the polymer *via* a hydrolysable Schiff base linkage by reacting amino groups of gentamicin (GEN) with aldehyde groups. Aldehyde groups can react rapidly with primary amines to yield a hydrolysable linkage,^36,62–66^ that allows a slow release of antibiotic in the middle acidic microenvironment of biofilm.^67,68^ To confer water solubility to the polymers, we prepared an amphiphilic block copolymer, constituted of a hydrophilic block (POEGMA), which is closely related to polyethylene glycol leading to excellent biocompatibility, and a short hydrophobic block containing aldehyde groups (PVBA) for further conjugation with gentamicin.

### Synthesis of POEGMA-*b*-PVBA block copolymer

**Scheme 1:**
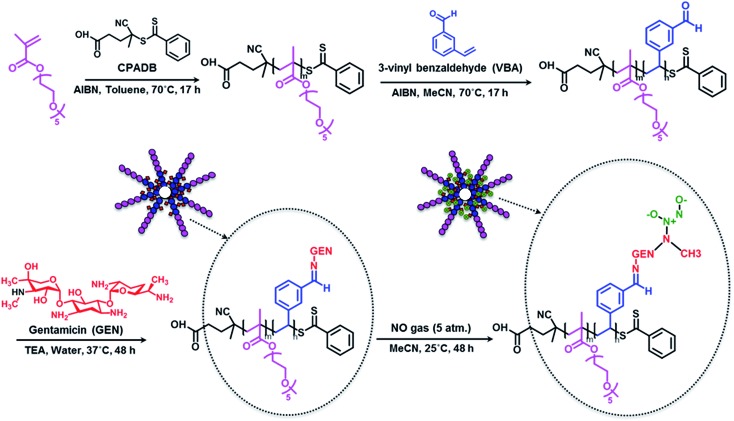
Schematic approach for the preparation of gentamicin-NONOate nanoparticles *via* RAFT polymerization.

Block copolymer POEGMA-*b*-PVBA was synthesized using living polymerization (*i.e.* reversible addition fragmentation chain transfer (RAFT) polymerization (Scheme 1). Poly((oligoethylene glycol) methyl ether methacrylate) (POEGMA) macro-RAFT agent was prepared in toluene at 70 °C in the presence of 4-cyanopentanoic acid dithiobenzoate (CPADB) as a RAFT agent and oligo(ethylene glycol) methacrylate (OEGMA) as monomer. The monomer conversion was monitored *via* ^1^H NMR spectroscopy by comparing the vinyl proton signals (at 6.1 and 5.6 ppm) with ester –OC*H*_2_ proton peaks (at 4.1 ppm). At ∼80% monomer conversion, the polymerization was stopped to avoid the formation of significant dead polymers; then, the polymer product was purified by several precipitations (three times) in petroleum spirits. The molecular weight obtained by SEC analysis is in good agreement with the theoretical value (*M*_n, theo_ = 10 800 g mol^–1^, *M*_n, SEC_ = 11 200 g mol^–1^, PDI = 1.08). Subsequently, POEGMA was successfully chain extended in the presence of 3-vinylbenzylaldehyde (VBA) to afford POEGMA-*b*-PVBA block copolymer. The conversion of VBA was determined to be around 50% using the vinyl signals at 5.0–6.0 ppm and aromatic signals at 6.5–7.5 ppm (ESI, Fig. S1†) to yield POEGMA_*x*_-*b*-VBA_*y*_, with *x* and *y* equal to 36 and 7. After purification, SEC analysis confirmed the successful chains extension by the molecular weight distribution shift to higher molecular weight (*M*_n, SEC_ = 13 700 g mol^–1^) and a low polydispersity index (PDI = 1.13) was obtained (ESI, Fig. S2†). ^1^H NMR and FTIR spectroscopy confirmed VBA incorporation by the presence of characteristic signals at 9.8 ppm and at 1710 cm^–1^ attributed to aldehyde group, respectively. The final copolymer was constituted by a longer block of OEGMA (36 units) to confer good solubility in water, and a shorter block of VBA (7 units) for functionality. This composition appears ideal to afford well-defined nanoparticles after conjugation with gentamicin. Indeed, if a longer block of VBA were employed, the copolymer would not be soluble in water.

### Conjugation of POEGMA-*b*-PVBA to gentamicin

**Fig. 1:**
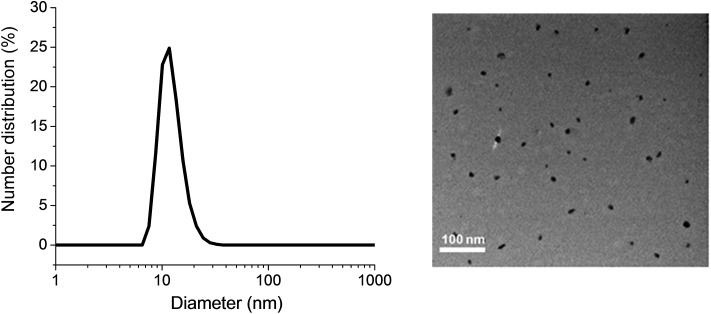
Dynamic light scattering (DLS) graph (left) and TEM image (right) depicting the size of POEGMA-*b*-PVBA-GEN nanoparticles.

Gentamicin was conjugated *via* a hydrolysable bond (Schiff base/imine) by reaction of primary amines with the aldehyde group of the copolymer for 48 h in the presence of triethylamine in water (pH = 8.0). Due to the presence of several amine groups per gentamicin, the hydrophobic segments in the core of the nanoparticles were simultaneously conjugated and cross-linked (Scheme 1). DLS results showed the number-average size of 15 nm and the polydispersity index (PDI) of 0.1, which is in good agreement of TEM data (Fig. 1). Volume and intensity distributions (Fig. S3†) were similar to the number distribution, indicating the nanoparticles were not forming aggregates. The resultant cross-linked polymer was further characterized by DMAc SEC (ESI, Fig. S4†). We observed a substantial shift in molecular weight of block copolymer after conjugation with gentamicin, from 13 700 g mol^–1^ to 110 000 g mol^–1^, which demonstrates the successful formation of cross-linked nanoparticles with a narrow polydispersity (PDI = 1.34). It should be noted that the SEC system was calibrated with linear polystyrene standards and the number average molecular weight obtained normally underestimates the actual molecular weight of the polymer. Sumerlin and co-workers have described a similar method for the synthesis of core cross-linked star polymers using difunctional organic compounds.^62,69–71^ After purification, the polymer was dissolved in acetonitrile in the presence of magnesium sulfate to remove water for further reaction with NO gas.

**Fig. 2:**
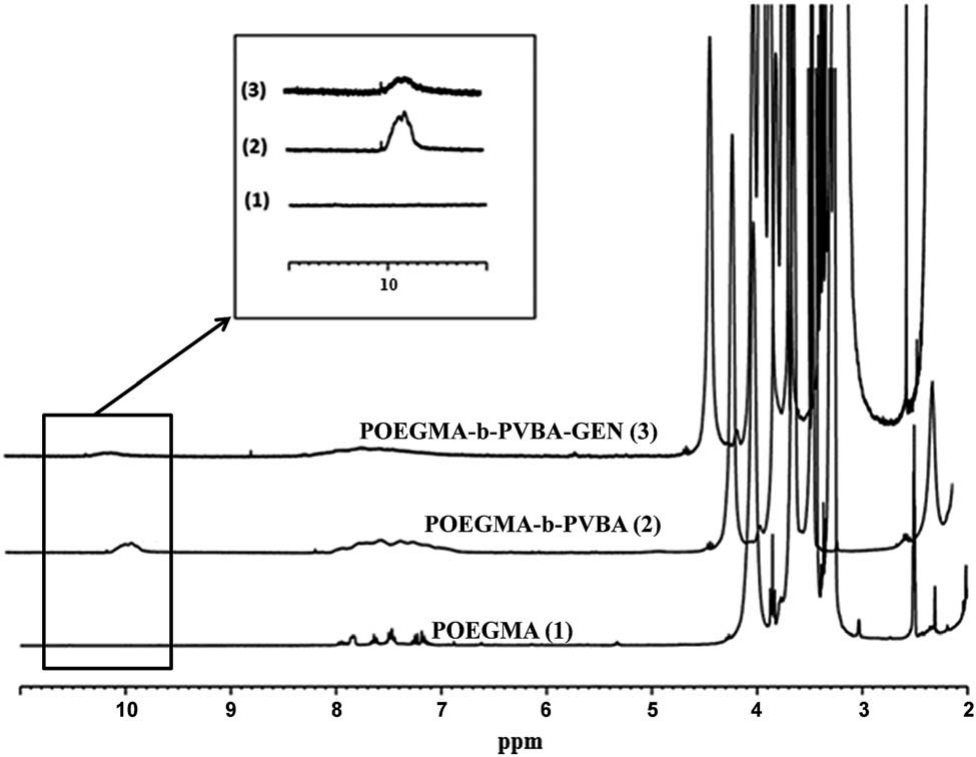
^1^H NMR spectra of purified POEGMA-*b*-PVBA-GEN overlaid with POEGMA-*b*-PVBA and POEGMA.

**Fig. 3:**
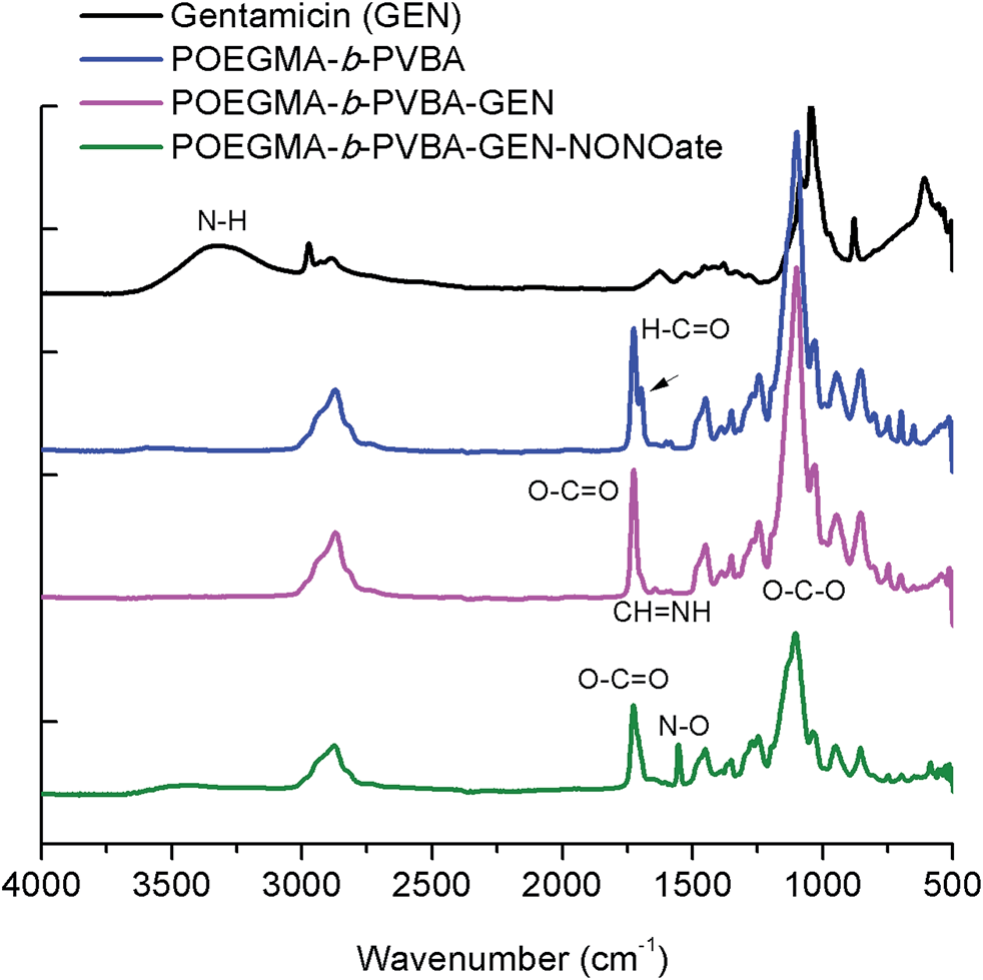
ATR-FTIR spectra of POEGMA-*b*-PVBA-GEN-NONOate compared with POEGMA-*b*-PVBA-GEN, POEGMA-*b*-PVBA and gentamicin.

The successful attachment of gentamicin was also confirmed using ^1^H NMR, elemental analysis and ATR-FTIR analyses. The conversion of aldehyde was monitored by NMR that following the decrease in the signal at 9.8 ppm (Fig. 2 and 3), which corresponds to the aldehyde group. A conjugation efficiency of 72% was determined by comparing the intensity of aldehyde signal at 9.8 ppm with the –CH_2_O ester of OEGMA at 4.1 ppm before and after the reaction with gentamicin after 48 h. The reaction time was extended up to 72 h, but the conjugation efficiency was not improved. This result could be attributed to the steric hindrance of gentamicin limiting its reactivity with aldehyde. After purification by precipitation, the polymer was dissolved in D_2_O and analyzed by ^1^H NMR (600 MHz) (ESI, Fig. S5†). We exploited the characteristic signal at 5.8 ppm which corresponds to –CH of gentamicin to determine the amount of gentamicin in polymer by comparing with the –CH_2_O ester of OEGMA at 4.1 ppm. NMR results showed around 2.3 gentamicins per polymer chain. However, this number of gentamicin can be underestimated due to the encapsulation of gentamicin in the core of the nanoparticles, which could lead to the incomplete solvation. Such behavior has been observed in previous studies by us^36,63^ and others^69,71^ for various systems. We have further analyzed the composition of the polymer by elemental analysis using X-ray Photoelectron Spectroscopy (XPS). Gentamicin contains five nitrogen atoms (from amine groups), while the polymer is only constituted by oxygen and carbon. RAFT agent contains only one nitrogen atom. By comparing the nitrogen amount before and after conjugation with gentamicin, we were able to estimate that 2.8–3.2 gentamicins where incorporated in the polymer (ESI, Table S2†). This value is relatively close with NMR data. According to the values obtained by aldehyde conversion from ^1^H NMR (72%, corresponding to 5 reacted aldehyde groups) and the number of gentamicin (3 units) per polymer chain, we were able to calculate that, on average, three gentamicin molecules reacted with 3 aldehyde groups of the block copolymer and the other two aldehyde groups reacted with additional amine groups from these gentamicin molecules. This could explain the formation of cross-linked nanoparticles observed by SEC analysis.

Finally, the conjugation of the copolymer to gentamicin was confirmed by ATR-FTIR spectroscopy. After conjugation with gentamicin, we noted a decrease in signals from the aldehyde bond at 1710 cm^–1^ and the presence of new absorption at ∼1620 cm^–1^ consistent with the formation of imine (Fig. 3).

### Post-modification of gentamicin conjugated POEGMA-*b*-PVBA nanoparticles with nitric oxide

As mentioned earlier, the aim of this study was to develop a nanocarrier system to disperse bacterial biofilms and kill bacteria in planktonic form. Our approach was to develop a compound that could disperse the biofilm, thereby making the bacteria more susceptible to antimicrobial agents, and then simultaneously treat the planktonic bacteria. In this study, gentamicin was chosen for this dual purpose owing to the presence of both primary and secondary amine groups. These functional groups allow for an easy conjugation of gentamicin to aldehyde functionalized polymers and the formation of gentamicin-*N*-diazeniumdiolate (gentamicin-NONOate) conjugated polymers by reaction of the secondary amine with nitric oxide (NO) gas. This is a novel approach, which combines the benefit of NO to the existing antibiotics. The NONOate group in gentamicin-NONOate complex can slowly release NO to re-generate native gentamicin. As gentamicin contains one secondary amine group, theoretically, one NONOate group could be attached per gentamicin. After purification, the polymeric nanoparticles were analyzed by three different techniques: UV-Vis spectroscopy, elemental analysis and ATR-FTIR. Firstly, UV-Vis was performed to quantify the amount of NONOate group by comparing the signal centered at 250 nm (ESI, Fig. S6†) before and after NO treatment using molar extinction coefficient for NONOate of 8500 M^–1^ cm^–1^.^59,72^ The UV-Vis after NO treatment shows an increased signal at around 250 nm, which demonstrates the successful attachment of NO. The amount of NONOate calculated by UV-Vis was close to one NONOate per gentamicin, which is in agreement with the expected values, *i.e.* 3 NONOate per polymer chain. Secondly, elemental analysis was carried out to quantify the amount of nitrogen. After NO treatment, we observed a significant increase of nitrogen (ESI, Table S2†), which corresponds to one NONOate per gentamicin, *i.e.* 3 NONOate per polymer chain. Both UV and elemental results are in good agreement. Finally, ATR-FTIR analysis confirmed the presence of the N–O band at 1510 cm^–1^ (Fig. 3), indicating the successful attachment of the NONOate group on gentamicin.

### Determination of gentamicin and nitric oxide release

The imine bond has previously been employed by our group and others for drug conjugation^62–66,69,70^ owing to its ability to slowly hydrolyze, which allows a sustainable release of therapeutic compounds. Bacterial biofilms and infected tissue by bacteria usually present a slight acidic pH (typically between 5.5–7.2), which should favor the release of gentamicin from the nanoparticles.^68,73^ Gentamicin conjugated nanoparticles were incubated in both pH 7.4 (phosphate buffer) and pH 5.5 (acetate buffer) (ESI, Fig. S7 and S8†). Nanoparticles were placed in a dialysis membrane with MWCO 3500 Da and the samples were taken at different time points for gentamicin and NO release. The gentamicin release kinetic from POEGMA-*b*-PVBA nanoparticles was monitored by comparing the aldehyde proton –CHO peak at 9.8 ppm and the –CH_2_O-proton peaks at 4.1 ppm using ^1^H NMR analysis. As expected, the intensity of the signal at 9.8 ppm increased over time, indicating the release of gentamicin. The release rate of gentamicin at pH 5.5 was slightly faster than at pH 7.4. After 17 h, around 50% of gentamicin had been released in both pH values (ESI, Fig. S9†). The slow release of gentamicin is desirable as it allows a prolonged action for a long treatment. Concurrently with the release of the gentamicin, the cross-linked structure disassembled into free block copolymer as shown by a decrease of molecular weight by SEC (ESI, Fig. S4 and Table S1†).

NO release from GEN-NO nanoparticles was assessed by the Griess assay, which is commonly employed to monitor the cumulative release of NO by several groups^24,74–81^ (ESI, Fig. S10†) and by amperometric measurement (ESI, Fig. S11†) following a previous procedure established by us.^24^ Griess assay measures the accumulation of nitrite and nitrate in water due to the rapid oxidation of NO in aerobic conditions, while amperometric measurement measures the instantaneous release of NO. The media employed to determine the NO release can affect the measurement as demonstrated by Schoenfisch's group^82^ and Reynolds' group.^77,82^ For this reason, we decided to perform the release in phosphate buffer, which has been demonstrated to give more accurate results.

**Fig. 4:**
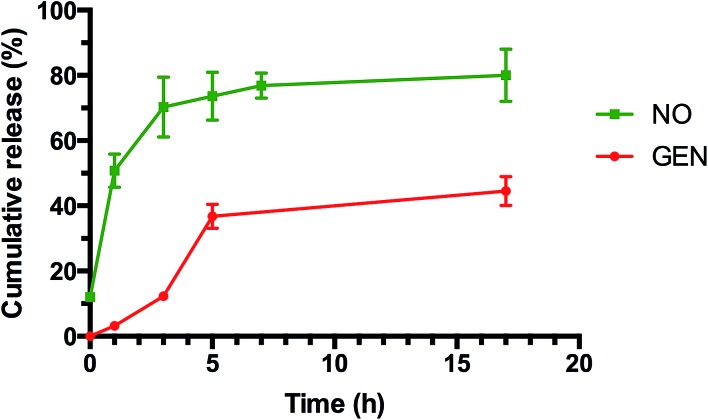
Cumulative release of NO and GEN from GEN-NO nanoparticles at pH 7.4, 37 °C. The concentration of GEN-NO nanoparticles was 5 mg mL^–1^; experiments were performed in triplicate (the points represent the average of three values).

As indicated in Fig. 4 (and ESI, Fig. S10 and S11†), both tests showed a prolonged release of NO for several hours. Interestingly, NO released from GEN-NO nanoparticles followed first order kinetics that had a half-life of approximately 1 h at pH 7.4. After 5 h, over 75% of NO was released from the polymeric nanoparticles according to Griess assay (Fig. 4). Amperometric measurement (ESI, Fig. S11†) showed a rapid release of NO as the beginning of the experiment, which is consistent with Griess assay (*i.e.* approximately 10% of NO has been released after 10 min). More importantly, amperometric experiment showed a continuous release of NO for over 3.5 h. After 3.5 h, we added a free radical scavenger, *i.e.* 2-phenyl-4,4,5,5-tetramethylimidazoline-1-oxyl-3-oxide (PTIO), into the vial in order to confirm that the amperometric signals being observed were due to NO. The signal of NO rapidly decreased after addition of PTIO. Interestingly, the encapsulation of NONOate in the core of nanoparticles appeared to enhance the stability of NONOate. Indeed, NONOate compounds such as diethylamine NONOate and spermine NONOate have very short half-lives (*i.e.* few minutes) as NONOates can spontaneously decompose to release NO in the presence of water.^83^ This relative slow release is desirable for our application to achieve a long dispersion of biofilms and avoid a rapid reformation of biofilm, which allows the gentamicin to kill bacteria.

### POEGMA-*b*-PVBA-gentamicin-NONOate eradicates *P. aeruginosa* biofilms

**Fig. 5:**
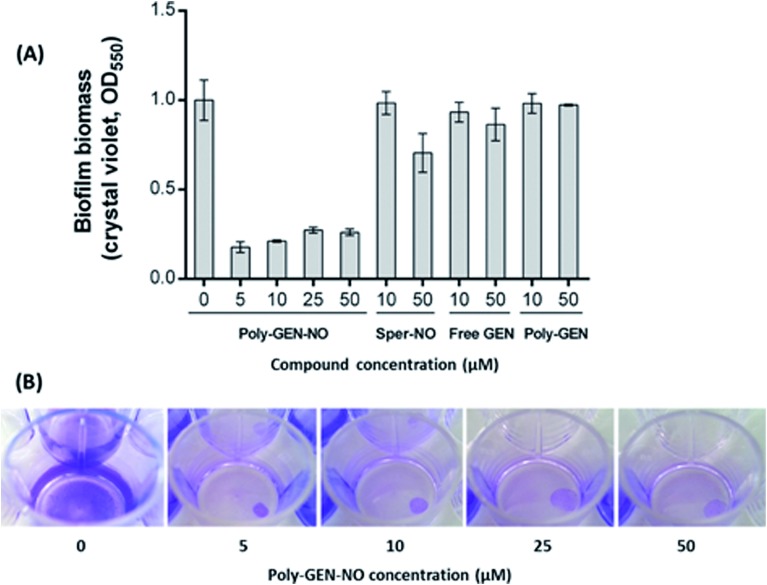
GEN-NO nanoparticles induced dispersal in *P. aeruginosa* biofilms. (A) Bacterial biofilms were grown in multi-well plates for 6 h in the absence of any treatment before being treated for a further 1 h with various concentrations (μM) of NO donor spermine NONOate (Sper-NO), free gentamicin or gentamicin-conjugated polymers (Poly-GEN) and GEN-NO nanoparticles (Poly-GEN-NO). Biofilm biomass was analyzed by crystal violet staining. Error bars represent standard error (*n* = 2). (B) Stained biofilms treated with the indicated concentrations of GEN-NO nanoparticles. Note: concentration based on GEN, one mole of GEN-NO nanoparticles is equivalent to one mole of Sper-NO and gentamicin.

To evaluate the effect of the new POEGMA-*b*-PVBA-gentamicin-NONOate (GEN-NO nanoparticles) on biofilms, we first tested their ability to release NO and disperse biofilms. Pre-established biofilms of the opportunistic pathogen and model biofilm-forming organism *P. aeruginosa* that had been grown for 6 h in the absence of any treatment, were treated with various compounds: (i) NO donor, spermine NONOate (Sper-NO); (ii) free GEN; (iii) gentamicin-conjugated polymers (Poly-GEN) and (iv) GEN-NO nanoparticles (Poly-GEN-NO). After 1 h treatment, the GEN-NO nanoparticles at 5 μM (based on GEN, one mole of GEN-NO nanoparticles is equivalent to one mole of Sper-NO and gentamicin) were found to induce biofilm dispersal, leading to 83% reduction in biofilm biomass as determined by crystal violet (CV) staining, compared with untreated control biofilms (Fig. 5). Increasing the nanoparticle concentrations to 10–50 μM (based on GEN), while still clearly inducing biofilm dispersal, resulted in slightly higher levels of staining on the well surfaces, which was possibly due to a higher amount of cells that were killed but not dispersed and thus also stained with CV. The addition of the NO donor, Sper-NO, which was used at equimolar concentrations compared to GEN-NO nanoparticles, led to only 30% reduction in biomass at 50 μM (Fig. 5). This result is comparable to other NONOate-conjugated polymers that were previously shown to disperse biofilms.^28,48^ Treatment with the antibiotic gentamicin alone only induced a small decrease in biofilm biomass at high concentrations, with 50 μM free gentamicin leading to less than 14% reduction. Gentamicin-conjugated polymers (*i.e.* without NO) did not reduce the amount of cells attached on the surface at all concentrations tested (Fig. 5).

**Fig. 6:**
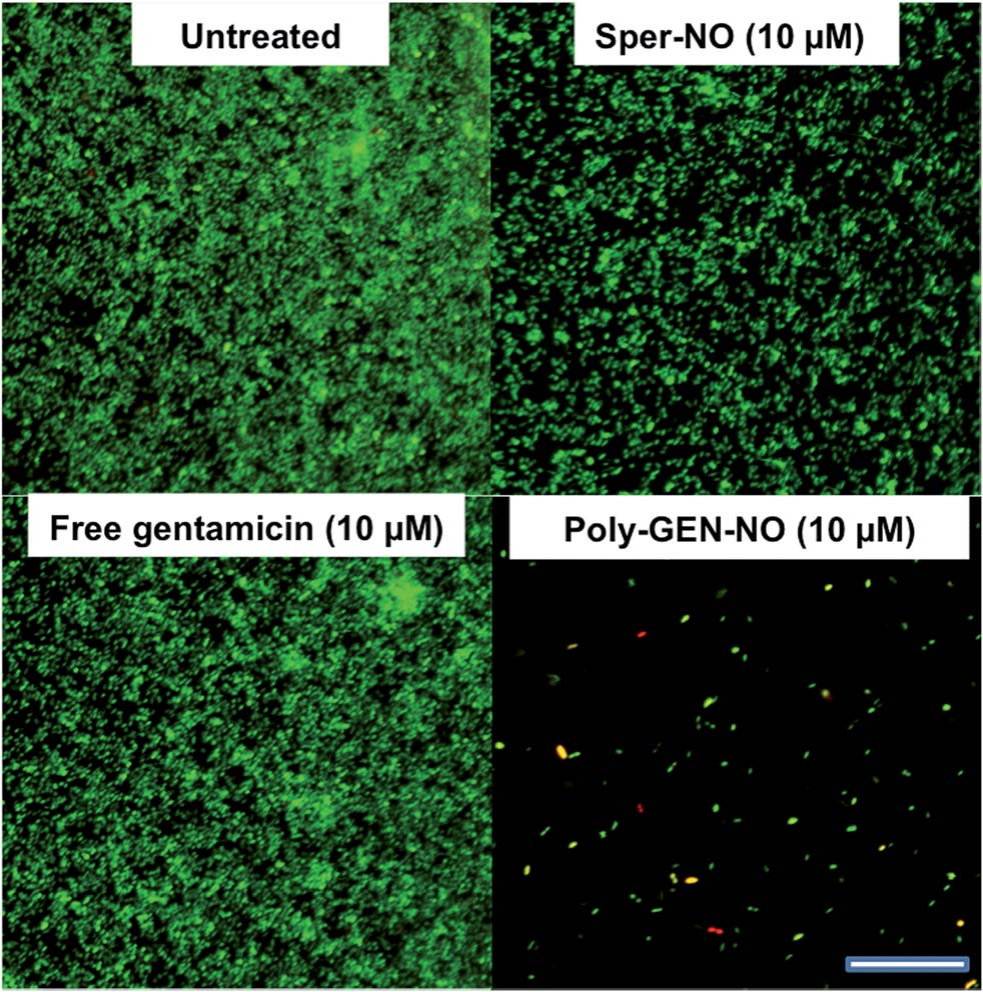
Representative confocal images showing *P. aeruginosa* biofilms stained with LIVE/DEAD kit. Biofilms were grown for 6 h and then treated with NO donor spermine NONOate (Sper-NO), free gentamicin, GEN-NO nanoparticles or left untreated for a further 1 h before staining. Viable and non-viable bacteria appear green and red, as well as those stained both green/red, respectively. Scale bar = 50 μm. Note: concentration based on GEN, one mole of GEN-NO nanoparticles is equivalent to one mole of Sper-NO and gentamicin.

Furthermore, confocal microscopy was used to evaluate the ability of the GEN-NO nanoparticles to disperse biofilms. Biofilm cells were stained with LIVE/DEAD dyes, where live and dead cells appear green and red, respectively. Cultures treated with GEN-NO nanoparticles at 10 μM displayed greatly reduced biofilm biovolume and exhibited more dead cells, compared with untreated control biofilms or those inoculated with the NO donor or gentamicin alone (Fig. 6). Overall, the crystal violet and confocal microscopy results confirmed that GEN-NO nanoparticles were able to release NO, which was made available to biofilms, and consequently, induced dispersal of biofilm cells.

**Fig. 7:**
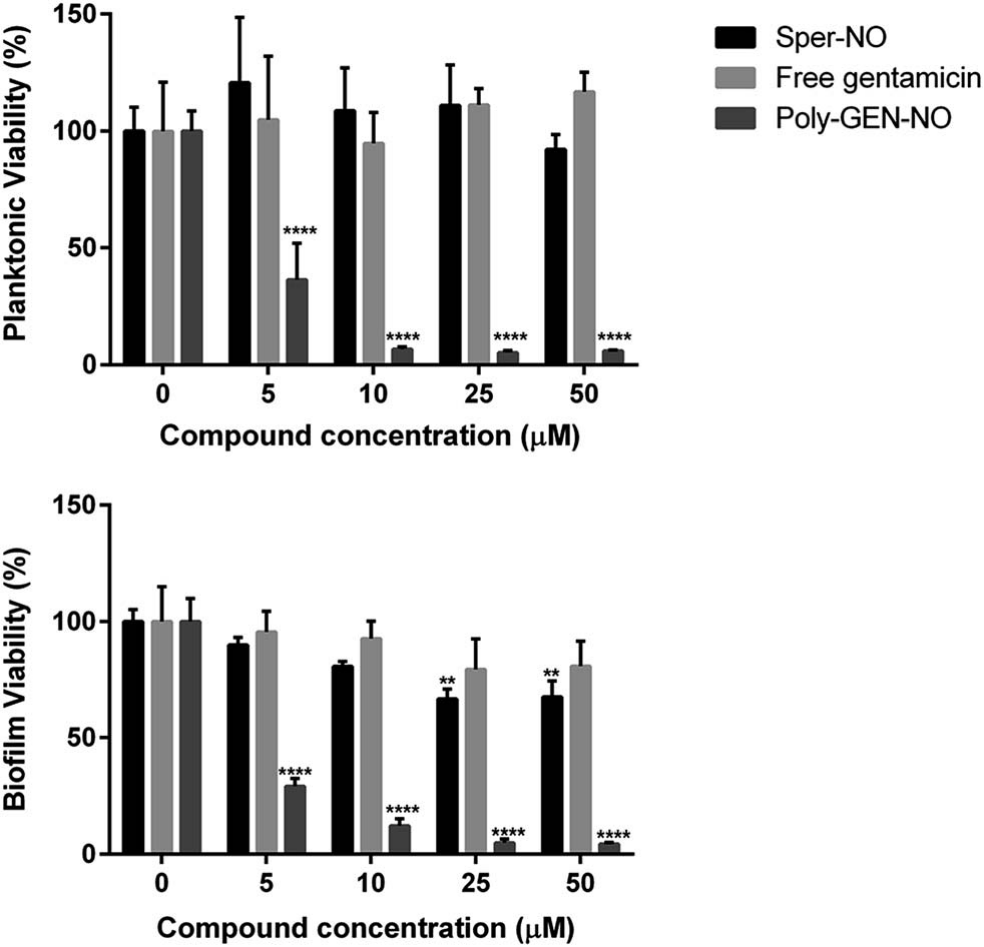
Effect of GEN-NO nanoparticles on *P. aeruginosa* viability after combined release of NO and gentamicin. *P. aeruginosa* biofilms were grown in multi-well plates for 6 h in the absence of any treatments and treated further for 1 h in the presence of 5–50 μM the NO donor spermine NONOate (Sper-NO), free gentamicin and GEN-NO nanoparticles (Poly-GEN-NO) before analyzing planktonic (top) and biofilm (bottom) viability by measuring the ATP content of bacteria. Error bars represent standard error (*n* = 4). Asterisks indicate statistically significant difference of treatments *versus* untreated culture (**, *P* < 0.01; ****, *P* < 0.0001). Note: concentration based on GEN, one mole of GEN-NO nanoparticles is equivalent to one mole of Sper-NO and gentamicin.

Next, the bactericidal properties of GEN-NO nanoparticles were investigated. *P. aeruginosa* biofilms were grown *in vitro* for 6 h as described above before being exposed to various treatments, including the NO donor, free GEN and GEN-NO nanoparticles. Then instead of analyzing the biofilm cultures by crystal violet staining, which can only account for total biomass, the viability of the cultures was assessed by measuring the ATP content of both biofilm and planktonic cells (Fig. 7). After 1 h treatment, a strong killing effect was observed in cell cultures treated with GEN-NO nanoparticles at 5–50 μM, compared with the untreated control, free gentamicin or NO donor alone. At 10 μM, GEN-NO nanoparticles almost completely eradicated both biofilm and planktonic cells. The viability of bacteria decreased significantly by 90% and 94% (*P* < 0.0001) in the biofilm and planktonic phases, respectively, compared with untreated cultures (Fig. 7). In contrast, gentamicin alone at 10 μM induced only a 7% and 5% decrease in biofilm and planktonic viability, respectively. In the presence of higher concentrations (*i.e.* 25 or 50 μM), gentamicin showed a slight increase in the reduction of biofilm cells, by up to 20%, but gentamicin did not affect planktonic viability (Fig. 7).

These results appeared consistent with previously published data, where a minimum concentration of 100 μM gentamicin was needed to eradicate *P. aeruginosa*.^84,85^ Furthermore, the NO donor spermine NONOate alone at a concentration of 10 μM, releasing an equivalent amount of NO compared to GEN-NO nanoparticles, did not display any toxicity towards planktonic cells (Fig. 7). Indeed, spermine NONOate induced an increase in cell viability by approximately 10% in the planktonic phase and a concomitant 19% reduction in the biofilm phase, indicative of dispersal events. At higher concentrations (*i.e.* 50 μM), the NO donor only caused a small and non-significant decrease in the planktonic phase, which was less than 8%. Results from the spermine NONOate cell viability assay indicated that the amount of NO in the GEN-NO nanoparticles was not involved in the killing effect of the compound, which was mainly due to the bactericidal activity of gentamicin. Taken together, these results strongly suggest that the combination of NO and gentamicin into a single polymeric structure leads to synergistic effects of biofilm dispersal and enhanced bactericidal activity and represents a highly promising strategy for combatting biofilm-related infections.

## Conclusions

In summary, we have synthesized a novel dual-action polymer based on an NO donor and the aminoglycoside gentamicin and demonstrated its potential for use in controlling *P. aeruginosa* biofilms. Combined and simultaneous delivery of NO and gentamicin is an attractive feature that would allow removing bacterial biofilms and killing the dispersed bacteria with one treatment. Encapsulated within the polymeric matrix the two agents are likely to have enhanced pharmacodynamic properties for systemic or local treatments. Furthermore, these compounds might be useful when applied as surface coating for the inhibition and prevention of biofilm formation on clinical surfaces or implants.
