# Supplementary material for: Co-delivery of nitric oxide and antibiotic using polymeric nanoparticles
Source: Chem Sci. 2015 Nov 10;7(2):1016–27. doi: 10.1039/c5sc02769a (PMC5531038; doi:10.1039/c5sc02769a)
Supplement: Supplementary file 1 [file SC-007-C5SC02769A-s001.pdf]

Electronic Supplementary Material (ESI) for Chemical Science.
This journal is © The Royal Society of Chemistry 2015

Electronic Supporting Information

Co-Delivery of Nitric Oxide and Antibiotic using Polymeric Nanoparticles

Thuy-Khanh Nguyen,[a] Ramona Selvanayagam,[a] Kitty K.K. Ho[b], Renxun Chen[b], Samuel Kutty,[b] Scott Rice[c,d], Naresh Kumar,[b] Nicolas Barraud,[c,e*] Hien T.T. Duong,[a#*] Cyrille Boyer[a*]

a) *Centre for Advanced Macromolecular Design (CAMD) and Australian Centre for NanoMedicine (ACN), School of Chemical Engineering, UNSW Australia, Sydney, NSW 2052, Australia*

b) *School of Chemistry, UNSW Australia, Sydney, NSW 2052, Australia*

c) *Centre for Marine-Innovation, School of Biological, Earth and Environmental Sciences, University of New South Wales, Sydney, Australia 2052*

d) *The Singapore Centre for Environmental Life Sciences Engineering and The School of Biological Sciences, Nanyang Technological University, Singapore*

e) *Department of Microbiology, Genetics of Biofilms Unit, Institute Pasteur, Paris, France*

f) Note : # *Present address: School of Chemistry, University of Sydney, Sydney, NSW 2052, Australia*

Supplementary Figures

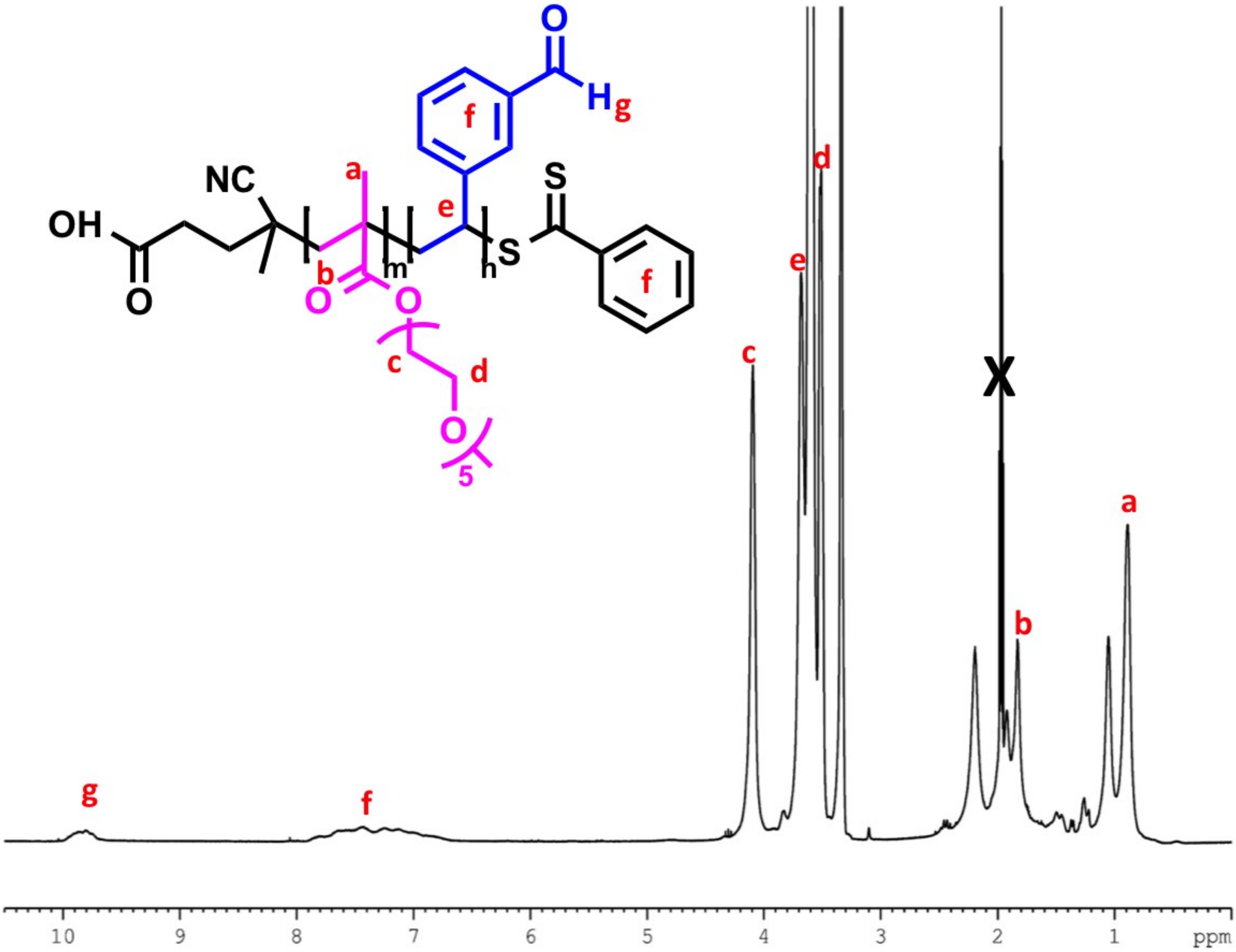

Figure S1. ^{1}H (300 MHz) NMR spectrum of purified POEGMA-*b*-PVBA in deuterated acetonitrile.

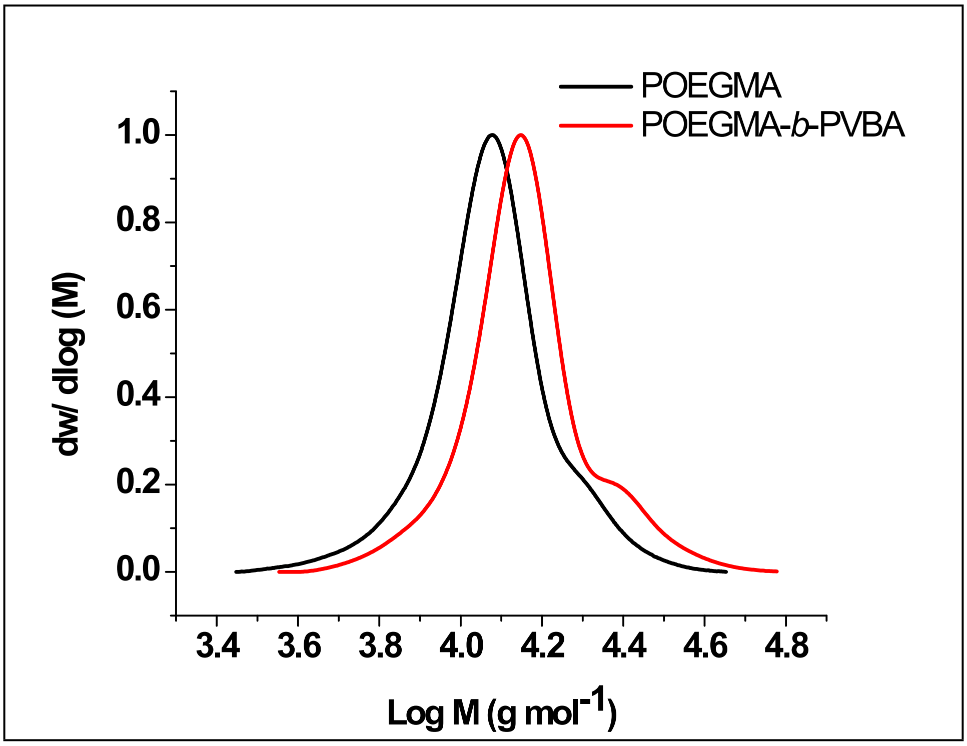

Figure S2. Molecular weight distribution of POEGMA and POEGMA-*b*-PVBA.

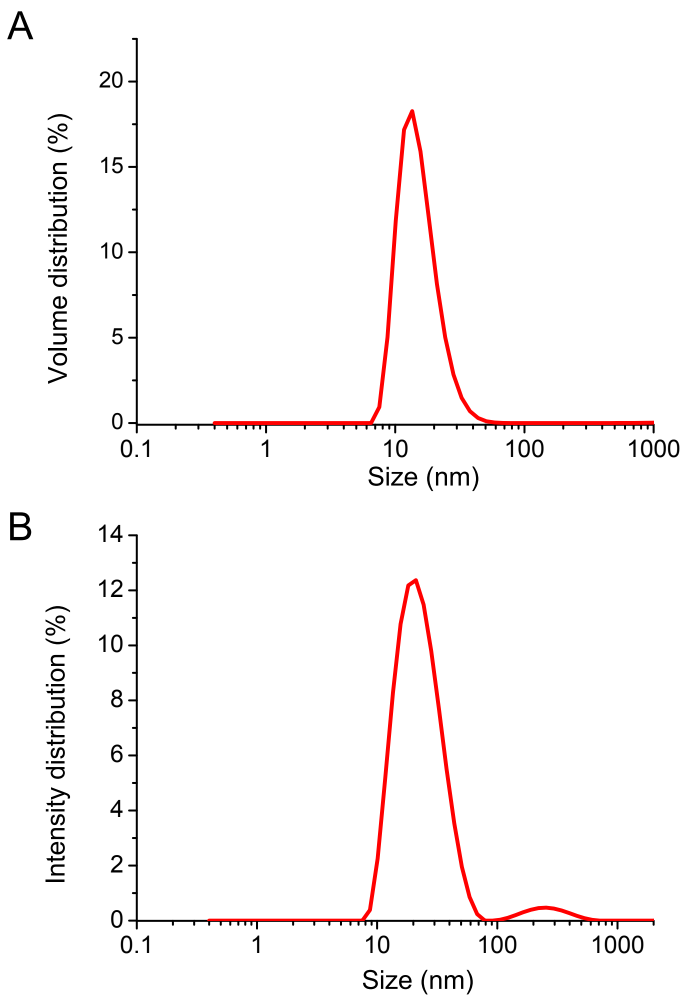

Figure S3. Dynamic light scattering (DLS) graphs of POEGMA-*b*-PVBA-GEN nanoparticles: A) volume distribution and B) intensity distribution.

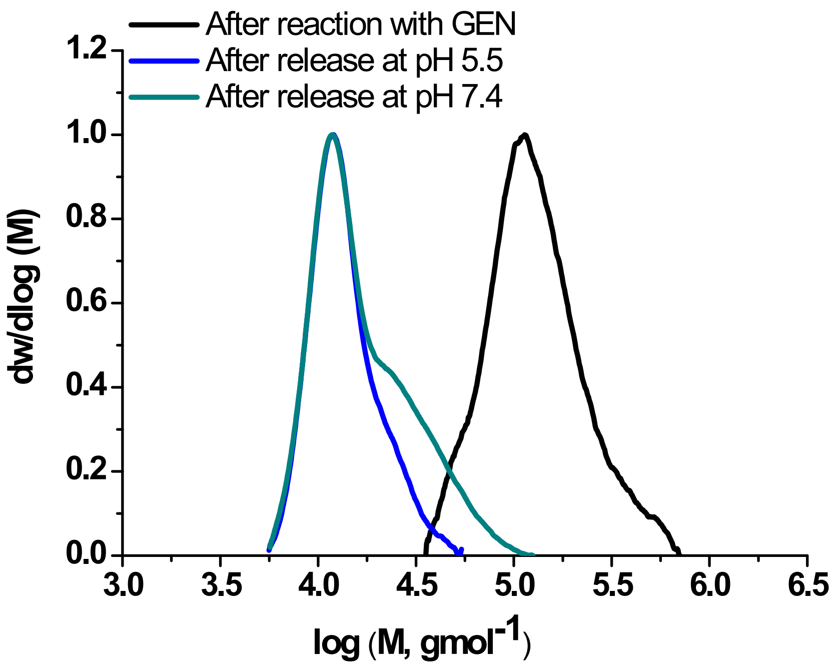

Figure S4. Comparison of molecular weight distributions of block copolymer after conjugation with gentamicin and NO, and polymers after gentamicin and NO released at pH 5.5 and pH 7.4.

Table S1. Molecular weight values and PDI of the polymers employed in this study.

Polymers	$M_{n,\ SEC}$ (g mol^{-1})	PDI
POEGMA	11200	1.08
POEGMA-*b*-PVBA	13 700	1.13
POEGMA-*b*-PVBA_GEN	110 000	1.34
POEGMA-*b*-PVBA_GEN-NONOate after release at pH 7.4	14 200	1.37
POEGMA-*b*-PVBA_GEN-NONOate after release at pH 5.5	12 500	1.18

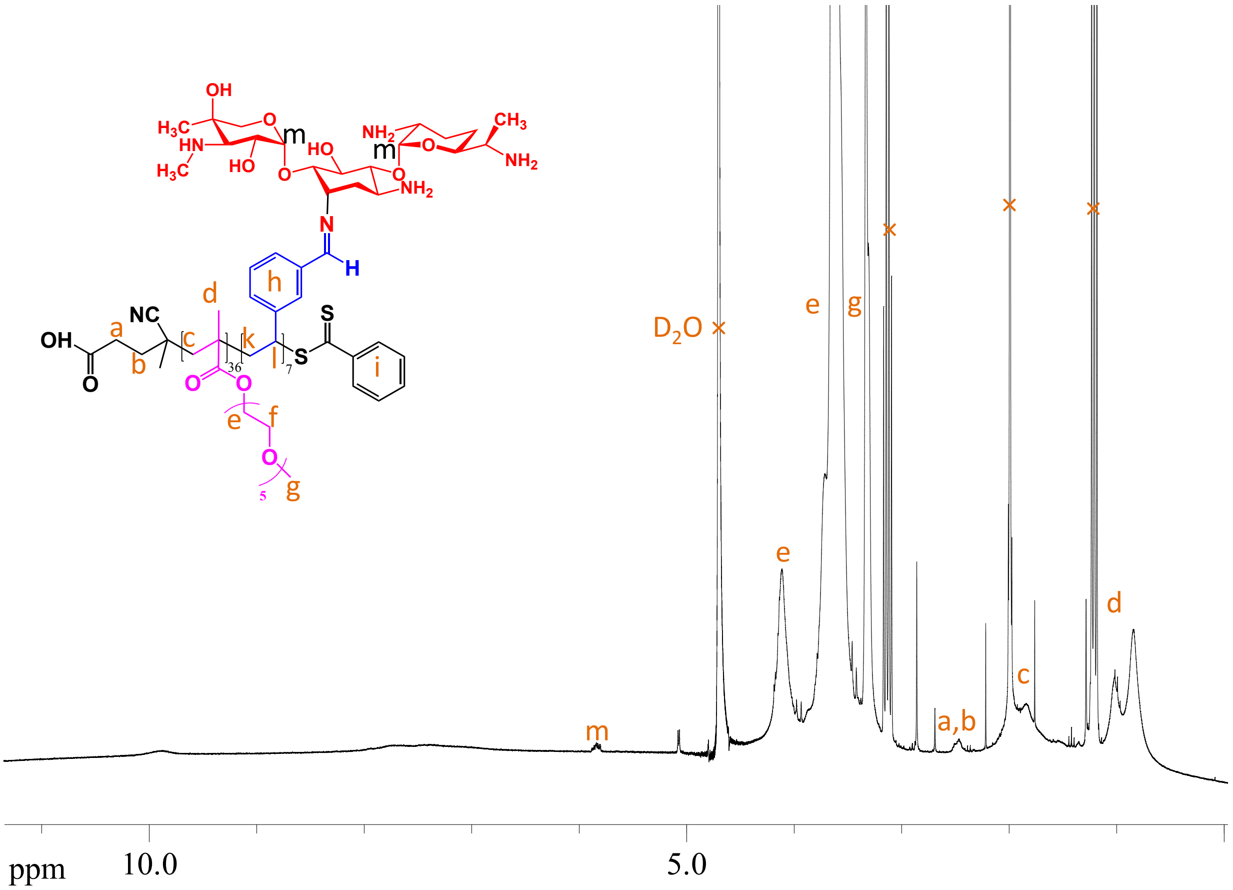

Figure S5. ^{1}H (600 MHz) NMR spectrum of purified POEGMA-*b*-PVBA-GEN polymers recorded in D_2O (1600 scans).

Number of gentamicin was calculated by using the following equation:

$N^{gentamicin} = [I^m/(I^e/DP^{OEGMA})]$

Where I^m, I^e and DP^{OEGMA} correspond to integral of m and e signals and degree of polymerization of OEGMA (36 in the polymer employed in this study).

Table S2. Elemental analysis of the polymers before, after gentamicin conjugation and after reaction with NO gas.

**Polymers**	Theoretical composition (Atomic-%)[a]				Experimental composition (Atomic-%)[b]				Number of Gentamicin	Number of NONOate
	C	O	N	S	C	O	N	S		
P(OEGMA)$_{36}$-*b*-P(VBA)$_7$	70.00	29.64	0.13	0.23	70.05	29.70	0.09	0.16	0	0
P(OEGMA)$_{36}$-*b*-P(VBA-GEN)$_7$	68.28	28.26	3.28	0.18	69.17	29.01	1.66	0.16	3 (±0.2)	0
P(OEGMA)$_{36}$-*b*-P(VBA-GEN-NO)$_7$	66.58	28.80	4.44	0.18	68.38	29.27	2.23	0.12	3 (±0.2)	3 (±0.2)

Note: a) theoretical values calculated using the following composition: P(OEGMA)$_{36}$-*b*-P(VBA)$_7$ and conjugation yield for GEN and NO ; b) average of values; c) number of gentamicin per macromolecule chains; d) number of NONOate per macromolecule chain.

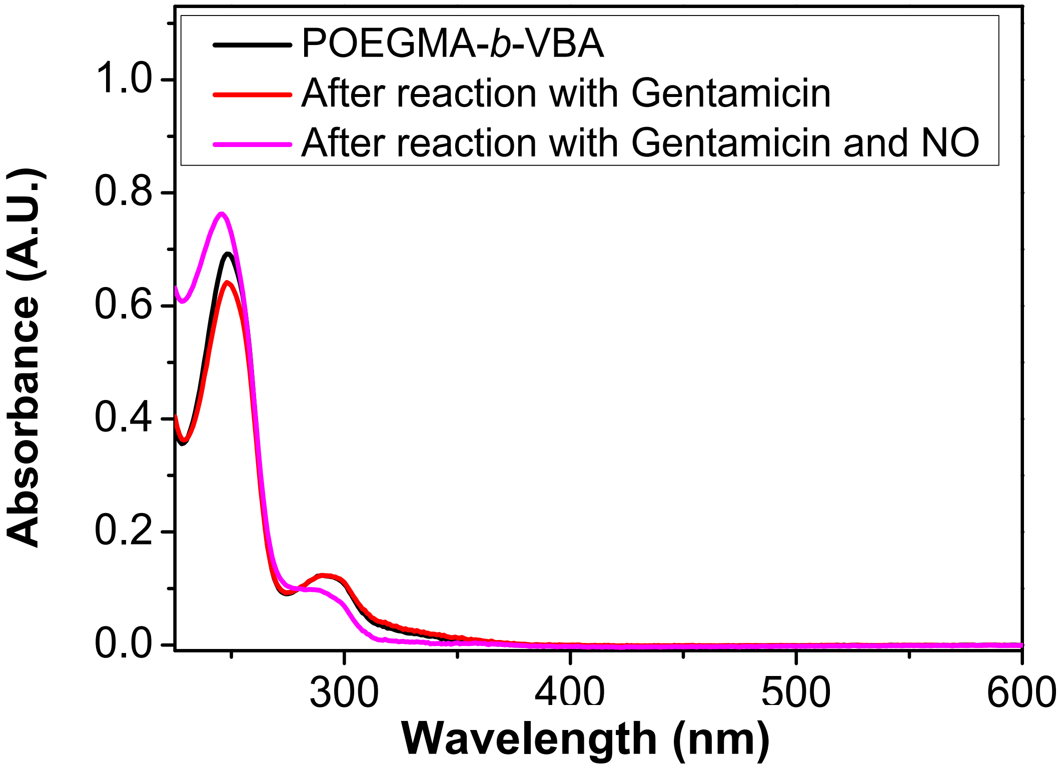

Figure S6. UV-Vis spectrum of POEGMA-*b*-PVBA-GEN-NONOate depicting successful NONOate conjugation. The spectra were recorded using the same concentration of polymer, 5 µM.

Number of mole of NONOate per mole of polymer was estimated by using the extinction coefficient of NONOate group at 250 nm (ε = 8 500 M^{-1} cm^{-1}),[1] using the following equation: $n^{NO} = ((A^{250nm\ after\ NO} - A^{250nm}_{gentamicin} / [Polymer]_0) \times l / \varepsilon$

Where, $A^{250nm\ after\ NO}$, $A^{250nm\ before\ NO}$, $[Polymer]_0$ and ε correspond to absorbance at 250 nm after NO and gentamicin conjugation, before NO and after gentamicin conjugation, polymer concentration, molar extinction coefficient of NONOate (ε = 8 500M^{-1} cm^{-1}) and length of the cuvette (1 cm). The measurement was repeated three times and represents an average of three measurements.

There are three NONOate groups (±0.1) per polymer chain.

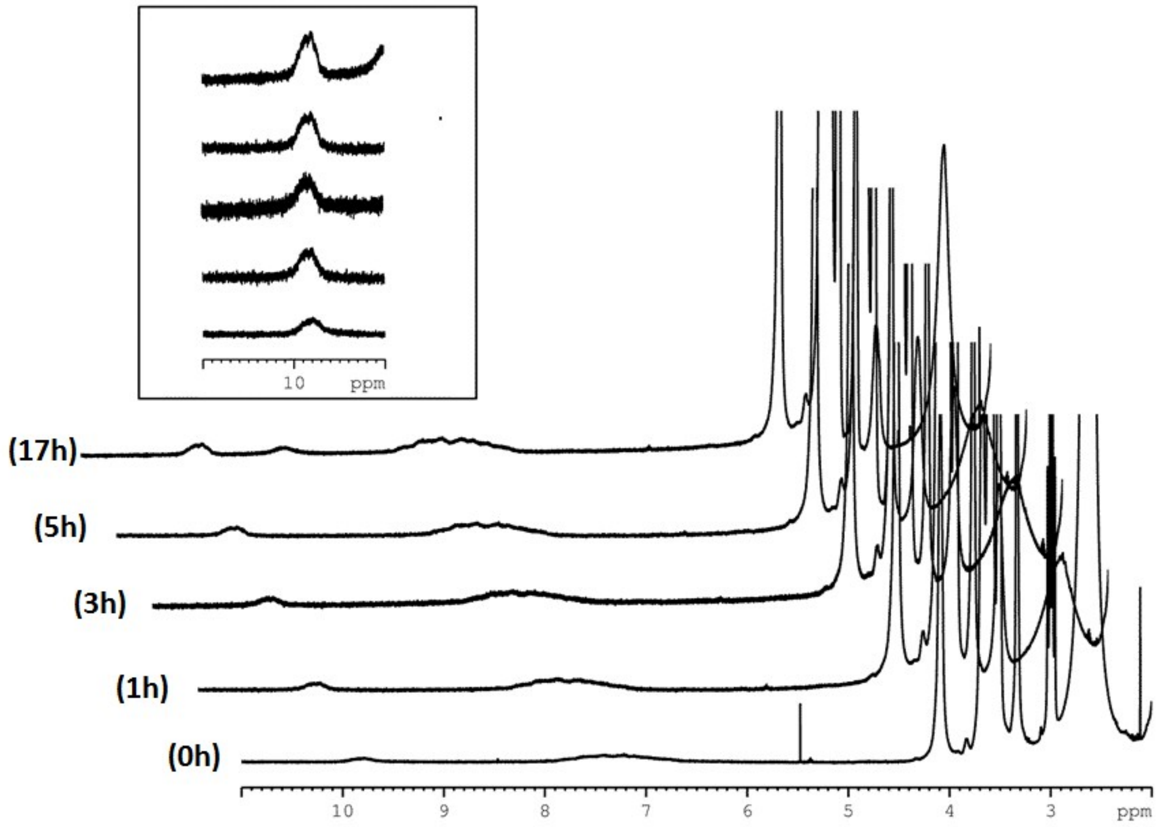

Figure S7. Stack plot of ^{1}H NMR (300 MHz) spectra of gentamicin conjugated POEGMA-*b*-PVBA nanoparticles incubated at pH 7.4 versus different time points (recorded in deuterated acetonitrile).

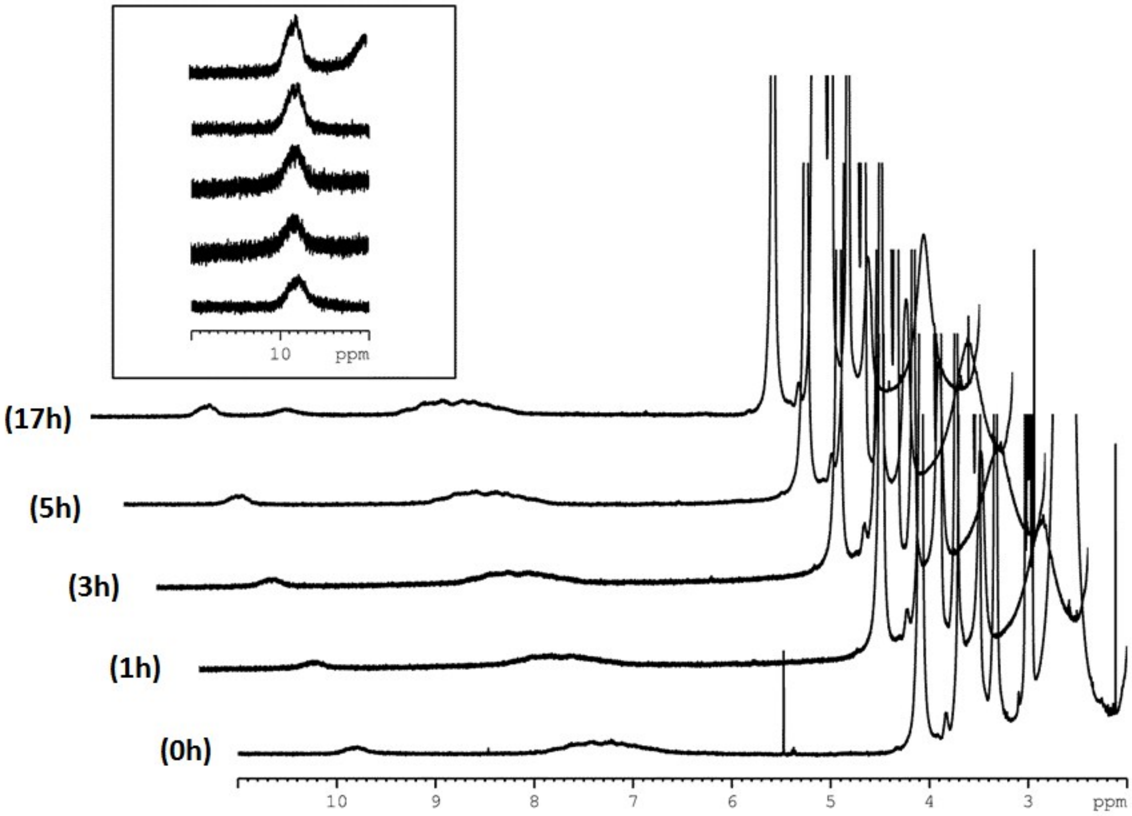

Figure S8. ^{1}H NMR (300 MHz) spectra of POEGMA-*b*-PVBA-GEN releasing gentamicin at pH 5.5 buffer solution at various time intervals recorded in deuterated acetonitrile.

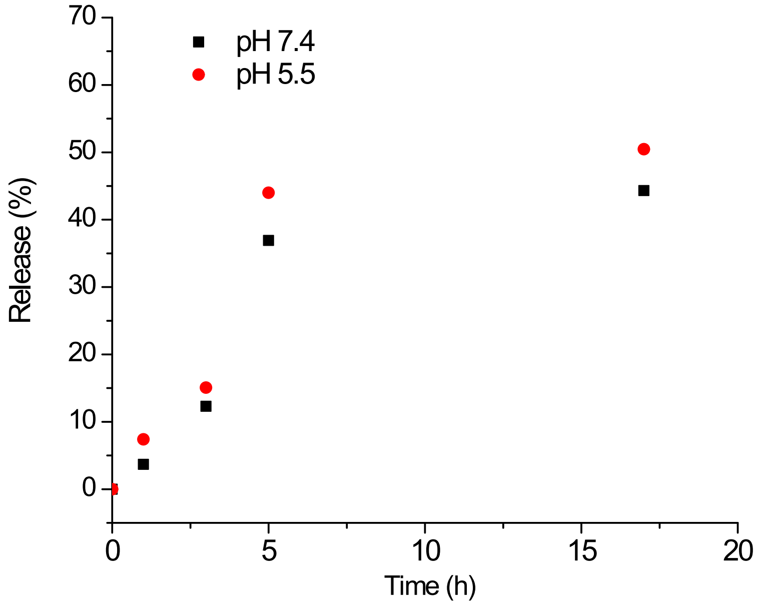

Figure S9. Release profile of gentamicin at predetermined time intervals in pH 7.4 and pH 5.5 buffer solutions

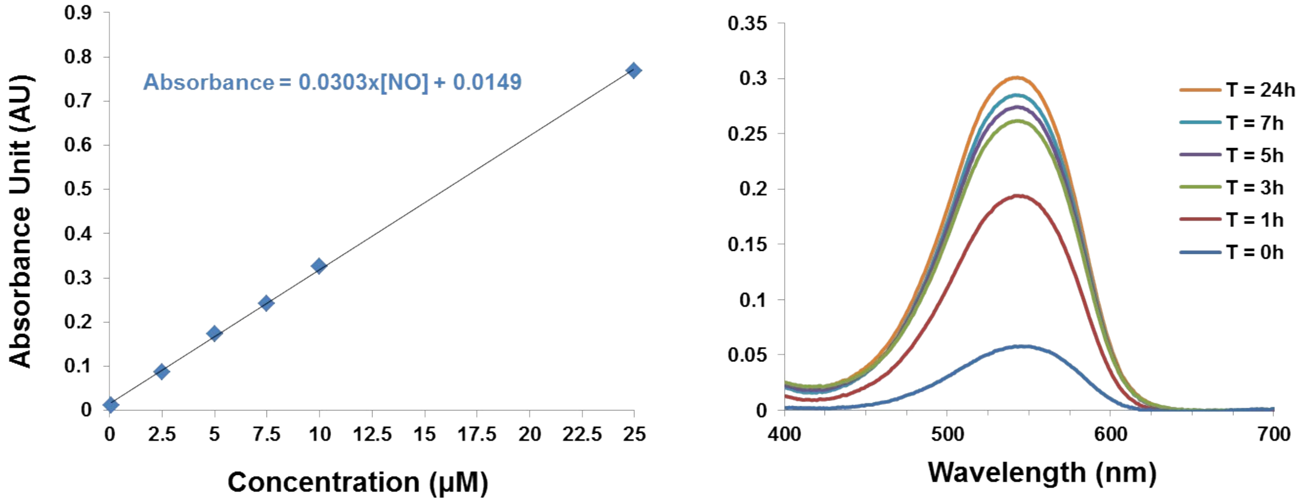

Figure S10. Calibration curve (left) and UV-Vis absorption (right) of the azo dye generated in the Griess assay at different concentrations of nitric oxide.

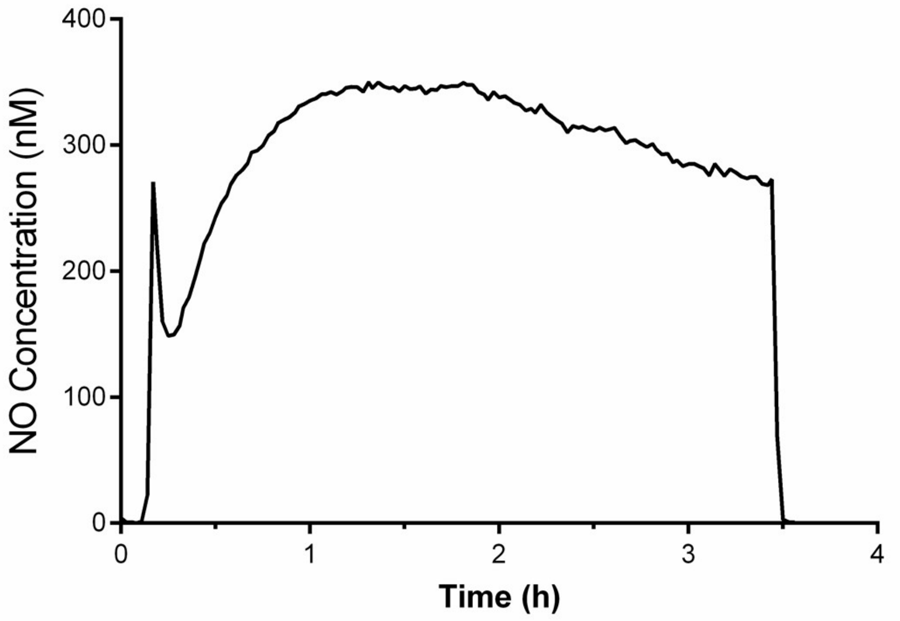

Figure S11. Amperometric characterization of NO release from 1mM GEN-NO nanoparticles. Amperometric measurment determines the instantenous amount of NO in solution. NO degrades rapidly in the presence of oxygen to yield nitrate and nitrite. The experiment was runned for 3.5 h, and showed a continuous release of NO during the period of time, which confirms the data obtained using Griess assay. Griess assay measures the accumulation of nitrate and nitrite in solution.

Additional References:

(1) R. Ferrero; F. Rodríguez-Pascual; M. T. Miras-Portugal; M. Torres, *British J. Pharmacology* **1999**, *127*, 779-787. C. M. Maragos; D. Morley; D. A. Wink; T. M. Dunams; J. E. Saavedra; A. Hoffman; A. A. Bove; L. Isaac; J. A. Hrabie; L. K. Keefer *J. Med. Chem.* 1991, **34**, 3242-3247.
